# Investigation and improvement of DNA cleavage models of polyamide + Cu(II) nuclease + OOH^- ^ligands bound to DNA

**DOI:** 10.1186/1472-6807-10-35

**Published:** 2010-10-17

**Authors:** Hongwei Yue, Yanyan Zhu, Yan Wang, Guangju Chen

**Affiliations:** 1 College of Chemistry, Beijing Normal University, Beijing100875, China

## Abstract

**Background:**

Copper nucleases as a famous class of artificial metallonucleases have attracted considerable interest in relation to their diverse potentials not only as therapeutic agents but also in genomic researches. Copper nucleases present high efficient oxidative cleavage of DNA, in which DNA strand scission occurs generally after hydrogen atom abstracted from a sugar moiety. In order to achieve the selective cleavage of DNA sequences by copper nucleases, the DNA specific recognition agents of the Dervan-type hairpin and cyclic polyamides can be considered as proper carriers of copper nucleases. Investigation of the DNA cleavage selectivity of copper nucleases assisted by the hairpin and cyclic polyamides at the molecular level has not yet been elucidated.

**Results:**

We carried out a series of molecular dynamics simulations for the nuclease [Cu(BPA)]^2+ ^or [Cu(IDB)]^2+ ^bound to the hairpin/cyclic polyamide and associated with DNA to investigate the selective DNA cleavage properties of Cu(II)-based artificial nucleases. The simulated results demonstrate that the DNA cleavage selectivity of the two nucleases assisted by the hairpin polyamide is improved efficiently. The [Cu(BPA)]^2+ ^or [Cu(IDB)]^2+ ^nuclease with a substrate OOH^- ^bound to the hairpin polyamide can be stably located at the minor groove of DNA, and possibly abstracts H atom from the sugar of DNA. However, the DNA cleavage properties of the two nucleases assisted by the cyclic polyamide are significantly poor due to the rigidity of linking region between the cyclic polyamide and nuclease. With introduction of the flexible linker -CH_2_CH_2_CH_2_NH_2_, the modified cyclic polyamide can assist the two copper nucleases to improve the selective DNA cleavage properties efficiently.

**Conclusion:**

A flexible linker and a proper binding site of the polyamide-type recognition agents play an important role in improving the DNA cleavage selectivity of copper nucleases. Current investigations provide an insight into the DNA cleavage specificities of chemical nucleases assisted by an appropriate nucleic acid recognition agent.

## Background

Artificial nucleases have attracted considerable attention for their diverse applications not only as therapeutic agents but also in biochemical research [1-4]. Among them, transition metal complexes have been studied extensively due to their diversity in structure and reactivity [5-8]. Copper complexes with the biological and accessible oxidative/reductive potentials have become a class of the most frequently studied artificial metallonucleases [9-16]. The first copper nuclease, [Cu(OP)_2_]^2+ ^(OP = 1,10-phenanthroline) [17,18], with high efficiency DNA cleavage property, motivated decade-long studies on mononuclear copper nucleases [3,5,19-22]. Especially, a great number of mononuclear copper nucleases[23-26] with BPA (BPA = bis(2-pyridylmethyl) amine)[27] and IDB (IDB = N,N-bis(2-benzimidazolylmethyl) amine)[28] ligands have attracted much attention due to their synthetic accessibility, low molecular weight and efficient DNA cleavage ability. Moreover, some experiments and theoretical calculations have predicted that the mononuclear copper nucleases present high efficient oxidative cleavage of DNA [7,23,29-33], in which DNA strand scission occurs generally after hydrogen atom abstractions of deoxyribose sugar moiety [2,27,34]. However, the oxidative DNA cleavage process only involves the hydrogen atom abstraction from any sugar in DNA, which makes most copper nucleases do not selectively recognize and cleave DNA sequences [35-37].

In order to improve the selective cleavage of copper nucleases to DNA molecules, specific DNA sequence recognition agents were introduced as carriers of copper nucleases [23,38]. In the case of DNA recognition agents, polyamides have presented promising characteristics due to its simple structure, synthetic accessibility and sequence specific affinity to base pairs in the DNA minor groove [39-42]. Recently, the experimental observations reported by Aldrich-Wright and co-workers demonstrated that the complexes of the Dervan-type polyamides combined with platinum compounds can successfully recognize the relevant sequences of DNA [43,44]. Moreover, among the Dervan-type polyamides, the hairpin and cyclic polyamides have presented higher affinities and specificities to DNA sequences than those of single-chain and antiparallel-double polyamides [1,40,45,46]. Specifically, eight-ring hairpin polyamide with the flexible β-alanine (*β*) residue that binds to DNA as a "turn-to-tail" model possesses excellent affinity and sequence specificity of DNA molecule [6,47]. The cyclic polyamides with *γ*-butyric acid (*γ*) can also efficiently recognize DNA sequences by a "turn-to-turn" model [48-50]. In the present work, we have performed a molecular dynamics (MD) study on the interactions of DNA with the ligands formed by each of the high efficient DNA-cleavage[22,51] copper nucleases, [Cu(BPA)]^2+ ^and [Cu(IDB)]^2+^, and by either the hairpin Dervan-type polyamide, ImImPyPy-γ-PyPyPyPy-β-Dp, or the cyclic Dervan-type polyamide, $\text{cyclo}[-\gamma -\text{ImPyPy}{-}^{NH_{2}}\gamma -\text{PyPyPy}-]$. Namely, six independent simulations were carried out, of which first four simulations were performed on hairpin-polyamide + [Cu(BPA)]^2+^- DNA, cyclic-polyamide + [Cu(BPA)]^2+^- DNA, hairpin-polyamide + [Cu(IDB)]^2+^- DNA and cyclic-polyamide + [Cu(IDB)]^2+^- DNA, to study the influences of different recognition agents and nuclease cleavage agents on the hydrogen abstractions from the sugar moiety of DNA; and the last two MD simulations were performed on modified-cyclic-polyamide $(\text{cyclo}{\left[-\gamma -\text{ImPyPy}{-}^{CH_{2}CH_{2}{\text{CH}}_{\text{2}}NH_{2}}\gamma -\text{PyPyPy}-])+[\text{Cu}\left(\text{BPA}\right)\right]}^{\text{2}+}–\text{DNA}$ and modified-cyclic-polyamide $(\text{cyclo}{\left[-\gamma -\text{ImPyPy}{-}^{CH_{2}CH_{2}{\text{CH}}_{\text{2}}NH_{2}}\gamma -\text{PyPyPy}-])+[\text{Cu}\left(\text{IDB}\right)\right]}^{\text{2}+}–\text{DNA}$ to investigate an improvement of the DNA cleavage selectivity of metal nucleases assisted by the modified cyclic polyamide with a flexible linker -CH_2_CH_2_CH_2_NH_2_.

## Methods

### Initial structures

The two polyamide-DNA systems are taken from the X-ray crystal structures of polyamide-DNA complexes. One is hairpin polyamide-DNA complex, ImImPyPy-γ-PyPyPyPy-β-Dp-d(AATATCCACCTGCA)_2 _(PDB code: 1M1A) [52]; and the other is cyclic polyamide-DNA complex, $\left(\text{cyclo}[-\gamma -\text{ImPyPy}-\gamma -PyPy{-}^{CH_{2}CH_{2}{\text{CH}}_{\text{2}}NH_{2}}\text{Py}-\right]$ - d(CGCTAACAGGC)_2 _(PDB code: 1PQQ) [53], assigned as HPD and CPD, respectively. The branch C atom of [Cu(BPA)]^2+ ^or [Cu(IDB)]^2+ ^nuclease was bound manually to the tail N atom (sp^3 ^hybridization) of each polyamide chain in HPD and CPD, which employed the protocols studied by Aldrich-Wright and co-workers for the polyamide - platinum(II) complexes [43,44]. Then the substrate OOH^- ^was introduced to the [Cu(BPA)]^2+ ^or [Cu(IDB)]^2+ ^nuclease to form an oxygen bridge for addressing the cleavage possibility. Finally, each whole system was explored to AutoDock 3.0 program[54] for selecting the initial structure for the MD simulation. However, the position of N-tail in CPD lies in the distal Py residue of cyclic polyamide, which makes the orientation of the linking site point away from the DNA. For selecting an appropriate linking site, the group of -NH_2 _(as a linker) on the C atom (sp^3 ^hybridization) of γ region was introduced to form the cyclic polyamide of $\text{cyclo}[-\gamma -\text{ImPyPy}{-}^{NH_{2}}\gamma -\text{PyPyPy}-]$ associated with DNA, assigned as CPD^*γ*^, which was suggested by the previous experimental observations [55,56]. Moreover, to improve the flexibility of liking region between the cyclic polyamide and nuclease, and to increase better the possibility of sugar hydrogen abstraction, the flexible linker of -CH_2_CH_2_CH_2_NH_2 _on the C atom (sp^3 ^hybridization) of *γ *region was introduced to form the modified cyclic polyamide of $\text{cyclo}[-\gamma -\text{ImPyPy}{-}^{CH_{2}CH_{2}{\text{CH}}_{\text{2}}NH_{2}}\gamma -\text{PyPyPy}-]$ associated with DNA, assigned as CPD^*γ*^Tail. The component sketches of six polyamide+copper nuclease+OOH^- ^ligands are shown in the additional files 1A, B, C, D, E and 1F. Therefore, HPD + [Cu(BPA)OOH]^+^, CPD^*γ *^+ [Cu(BPA)OOH]^+ ^and CPD^*γ*^Tail + [Cu(BPA)OOH]^+ ^were assigned as HPD-BPA, CPD^*γ*^-BPA and CPD^*γ*^Tail-BPA, respectively. Similarly, HPD + [Cu(IDB)OOH]^+^, CPD^*γ *^+ [Cu(IDB)OOH]^+ ^and CPD^*γ*^Tail + [Cu(IDB)OOH]^+ ^were assigned as HPD-IDB, CPD^*γ*^-IDB and CPD^*γ*^Tail-IDB, respectively. Given that each strand of DNA has some phosphate groups, sodium ion counterions were separately added to each system to achieve electroneutrality. The systems were solvated explicitly by using the TIP3P water potential inside a central simulation box. The box dimensions ensure the solvent shell extended to10 Å in all directions of each system studied.

### Molecular dynamics simulations

To take advantage of previous extensive simulations [44,54,57-60], the protocols employed therein were directly adapted in this study. The six MD simulations were carried out using the AMBER9 package[55] with the AMBER force fields of parm99[61,62] and gaff [63]. The atomic types for the studied polyamides, OOH^-^, [Cu(BPA)]^2+ ^and [Cu(IDB)]^2+^, except for copper atoms surrounding of [Cu(BPA)]^2+ ^and [Cu(IDB)]^2+ ^nucleases, were generated by using the ANTECHAMBER module included in AMBER9 program package. The force field parameters of copper center of [Cu(BPA)OOH]^+ ^and [Cu(IDB)OOH]^+ ^were evaluated based on the quantum chemical calculations in our previous work reported elsewhere [30,33,57]. The electrostatic potentials for RESP calculations have been calculated at the HF/6-31G** level of theory[64-66] using Gaussian03 program [67]. The RESP charges of the polyamides, OOH^-^, [Cu(BPA)]^2+ ^and [Cu(IDB)]^2+ ^were derived by the RESP program based on the calculated electrostatic potentials.

The protocol for all MD simulations was described herein as follows: (1) The systems were energetically minimized to remove unfavorable contacts. Four cycles of minimizations were performed as follows, i.e. 5000 steps of each minimization with harmonic restraints from 100, 75, 50 to 25 kcal·mol^-1^·Å^-2^, which means that the restraints were relaxed stepwise by 25 kcal·mol^-1^·Å^-2 ^per cycle, on DNA, polyamide and nuclease positions. The fifth cycle consists of 10000 steps of unrestrained minimization before heating process. The cutoff distance used for the nonbonded interactions was 10 Å. The SHAKE algorithm [68] was used to restrain the bonds containing hydrogen atoms. (2) Each energy-minimized structure was heated over 120 ps from 0 to 300 K (with a temperature coupling of 0.2 ps), while the positions of DNA, polyamide and nuclease were restrained with a small value of 25 kcal·mol^-1^·Å ^-2^. The constant volume was maintained during the processes. (3) The unrestrained equilibration of 200 ps with constant pressure and temperature conditions was carried out for each system. The temperature and pressure were allowed to fluctuate around 300 K and 1 bar with the corresponding coupling of 0.2 ps, respectively. For each simulation, the integration step of 2 fs was used. (4) Finally, production runs of 30 ns were carried out by following the same protocol. A time of 200 ps after thermal warm-up in each simulation was selected as a starting point for data collections. During the production run, 15000 structures for each simulation were saved for post-processing by uniformly sampling the trajectory.

## Results and Discussion

With the help of thirty nanosecond-long MD simulations, it was noted that these systems in water environment can be successfully simulated by using the protocol described above. The energies and root-mean-square deviations (RMSDs) for each simulation have been examined to clarify if each system had attained equilibrium. The RMSDs of each studied system with respect to its starting structure have attained equilibrium after 5 ns. The energies were found to be stable during the course of each remainder simulation. Hence, a time of 5 ns after thermal warm-up in each simulation was selected as a starting point for data collection and further analysis. The trajectory analysis for each system involves extracting the equilibrated conformations between 5 ns and 30 ns of simulation time, recording 12500 snapshots at every 2 ps time-interval of each trajectory. For the analyses of trajectories, the PTRAJ module of the AMBER9 program was used to extract production conformations.

### Simulations of polyamide + [Cu(BPA)OOH]^+^-DNA complexes (HPD-BPA and CPD^*γ*^-BPA)

**Figure 1:**
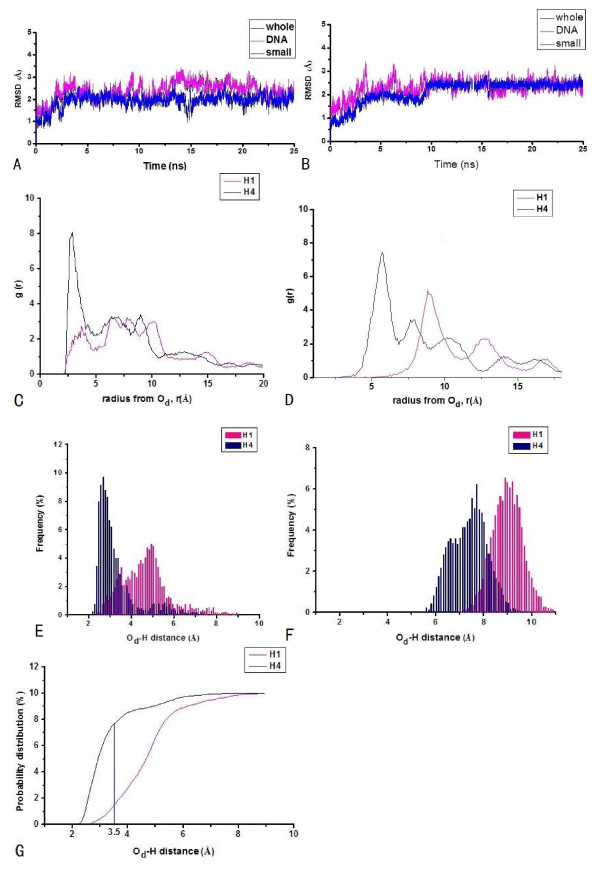
RMSDs with respect to the starting structures in the simulations of polyamide + [Cu(BPA)OOH]^+^-DNA (polyamide + [Cu(BPA)OOH]^+ ^-DNA (black), polyamide + [Cu(BPA)OOH]^+ ^(blue), and DNA alone (magenta)), RDF curves of the distal O_d _atom of OOH-C1'H atom(pink)/C4'H atom (navy) of sugars, probability distributions (the histogram bin width of 0.1 Å), and integration plot of probabilities of O_d_-C1'H (pink) and O_d_-C4'H (navy) distances: (A), (C), (E), (G) for hairpin polyamide + [Cu(BPA)OOH]^+^-d(AATATCCACCTGCA)_2 _(HPD-BPA) and (B), (D), (F) for cyclic polyamide + [Cu(BPA)OOH]^+ ^-d(CGCTAACAGGC)_2 _(CPD^*γ*^-BPA).

To investigate the DNA cleavage specificities of the metal nuclease assisted by different recognition agents, the [Cu(BPA)OOH]^+ ^ligand was bound to hairpin and cyclic polyamides, respectively. The two polyamide + [Cu(BPA)OOH]^+ ^- DNA complexes are assigned as HPD-BPA and CPD^*γ*^-BPA. The small RMSD values of these two systems predict that the interactions between the polyamide ligand and the DNA molecule in each system are strong enough to hold firmly the [Cu(BPA)OOH]^+ ^ligand in the minor groove of DNA. Namely, the average RMSD values of two systems derived from the simulations over 12500 structures in the computed trajectories for HPD-BPA and CPD^*γ*^-BPA are respective 2.38 and 2.31 Å, as shown in Figures 1A and 1B. The mean-square fluctuations correlated with temperature factors for the systems support that each system reaches a relatively stable situation with small RMSD changes during the course of the simulation. The radial distribution functions (RDF) between the O_d _atom of OOH^- ^ligand and the hydrogen atoms of sugar of DNA may supply primary information regarding DNA strand scission possibility. The RDFs, probability distributions and integration plots of probabilities of the O_d_-C1'H and O_d_-C4'H distances of the nearest cytosine and thymine bases, are shown in Figures 1C, E and 1G, respectively, for HPD-BPA. The RDFs and the probability distributions of the O_d_-C1'H and O_d_-C4'H distances of the nearest cytosine and thymine bases are shown in Figures 1D, and 1F, respectively, for CPD^*γ*^-BPA. Figures 1C and 1D show the first sharp RDF peak centered at 2.85 Å corresponding to a direct interaction of OOH^- ^with C4'H atom for HPD-BPA, and that centered at 5.98 Å with the C4'H atom for CPD^*γ*^-BPA, respectively. In addition, Figures 1E and 1F present the probability distributions of the O_d_-C4'H distance less than 3.5 Å with 76.5% of the simulation time versus those of the O_d_-C1'H distance less than 3.5 Å with 16.5% of the simulation time for HPD-BPA, and the probability distributions of the O_d_-sugar hydrogen atom distances less than 3.5 Å all with 0% of simulation times for CPD^*γ*^-BPA, respectively. The integration plots of probabilities in Figure 1G show that the C4'H abstraction is more favorable than the C1'H one for HPD-BPA. However, Figure 1F presents the properties of the poor hydrogen abstractions from the sugars of DNA for CPD^*γ*^-BPA.

**Figure 2:**
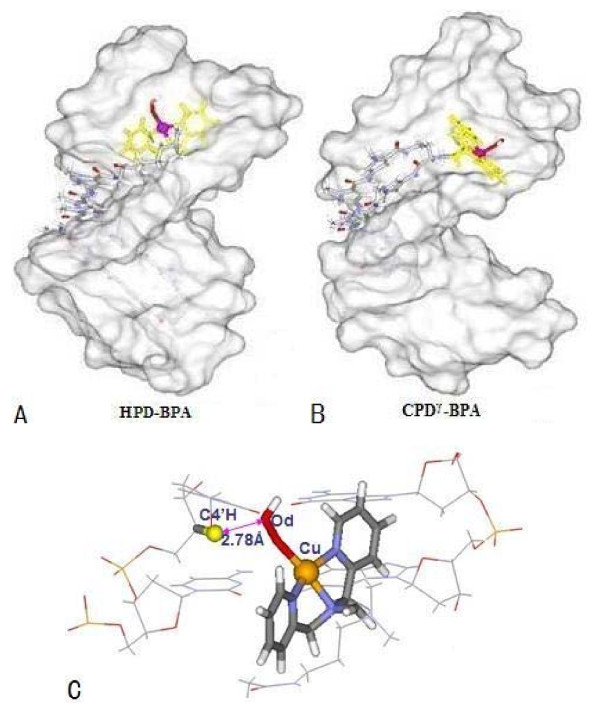
**Space-filling DNA models of polyamide + [Cu(BPA)OOH]^+ ^ligand binding to the minor groove of DNA; (A) for hairpin polyamide + [Cu(BPA)OOH]^+^-d(AATATCCACCTGCA)_2 _(HPD-BPA); (B) for cyclic polyamide + [Cu(BPA)OOH]^+ ^-d(CGCTAACAGGC)_2 _(CPD^*γ*^-BPA); (C) average structure of hairpin polyamide + [Cu(BPA)OOH]^+ ^ligand orientating to C4'H atom of sugar.**

Analyses of the average structures of the trajectories obtained from the simulations of HPD-BPA and CPD^*γ*^-BPA reveal spatial details of the interactions of the entire ligands with DNA molecules, which supports the general observations described above. Specifically, (1) for HPD-BPA, the cleavage agent of [Cu(BPA) OOH]^+ ^is located at the minor grove of DNA during the course of the simulation; the equatorial plane of the [Cu(BPA)]^2+ ^nuclease is parallel to the wall of the minor groove of DNA with the angle of 12° between the plane of nuclease and the wall of minor groove, as shown in Figure 2A. However, for CPD^*γ*^-BPA, the cleavage agent of [Cu(BPA) OOH]^+ ^cannot remain in the minor groove, whose orientation is removed from the minor groove of DNA, resulting from the rigidity of the N-tail of cyclic polyamide that constrains the flexibility of the [Cu(BPA)]^2+ ^nuclease, as shown in Figure 2B. (2) The chains of polyamide for each system also remain almost parallel to the wall of the minor groove of DNA; the N-H groups nearby Im/Py of polyamides play an important role in acting as H-bonds donors to the N or O atoms of nucleobases located on the floor of the minor groove of DNA. Moreover, the H atom of the branch C atom in [Cu(BPA)]^2+ ^and the N or O atom of bases of neighboring DNA can also form H-bonds. The affinity of polyamides with DNA are enhanced mainly by an increase in the number and strength of observed H-bonds between the polyamide-nuclease ligand and the DNA molecule, i.e., the H-bonds formed between the C-H groups of [Cu(BPA)]^2+ ^and O4 atom of T8 base in DNA with C-O distance of about 3.1 Å. (3) The average structure of each system presents a relatively stable spatial conformation during the course of simulation. Figures 2A and 2B show the average structures from the simulations for HPD-BPA and CPD^*γ*^-BPA, respectively. The average structure with the average O_d_-C4'H distance of 2.78 Å for HPD-BPA in Figure 2C predicts the DNA cleavage possibility at the tail region of the polyamide, because that the Cu atom of [Cu(BPA)]^2+ ^approaches the nearest sugar C4'H of C10 base. However, from Figure 2B for CPD^*γ*^-BPA, it is obvious that the structural characteristics reveal theoretically the poor cleavage ability of [Cu(BPA)]^2+ ^nuclease due to the restraint of the rigid linker of the cyclic polyamide bound to the [Cu(BPA)]^2+ ^nuclease. Namely, the restraint of DNA cleavage of the [Cu(BPA)]^2+ ^nuclease results from the short linker between the branch C atom of nuclease and backbone of cyclic polyamide.

### Simulations of polyamide + [Cu(IDB)OOH] ^+ ^- DNA complexes (HPD-IDB and CPD^*γ*^-IDB)

**Figure 3:**
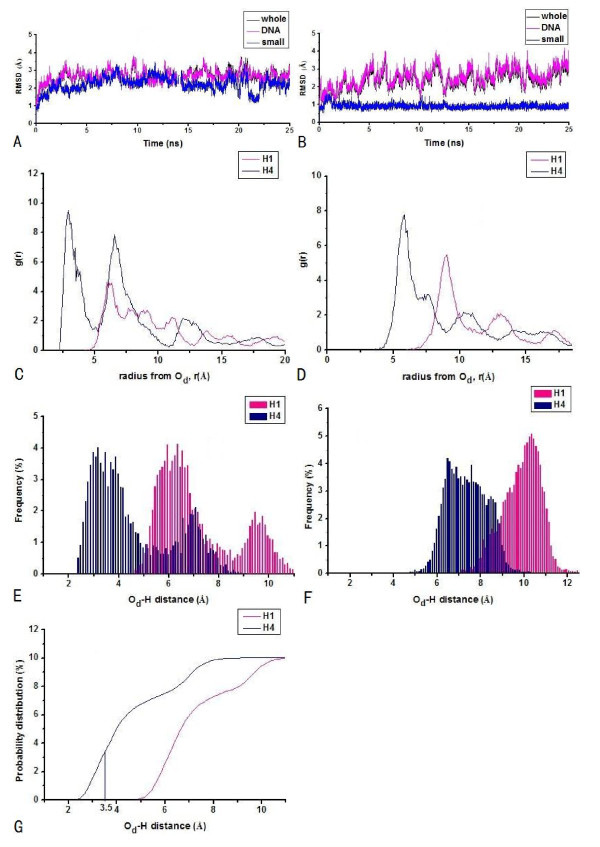
**RMSDs with respect to the starting structures in the simulations of polyamide + [Cu(IDB)OOH]^+^-DNA (polyamide + [Cu(IDB)OOH]^+^-DNA (black), polyamide + [Cu(IDB)OOH]^+ ^(blue), and DNA alone (magenta)), RDF curves of the distal O_d _atom of OOH-C1'H atom (pink)/C4'H atom (navy) of sugars, probability distributions (the histogram bin width of 0.1 Å), and integration plot of probabilities of O_d_-C1'H (pink) and O_d_-C4'H (navy) distances: (A), (C), (E), (G) for hairpin polyamide + [Cu(IDB)OOH]^+ ^-d(AATATCCACCTGCA)_2 _(HPD-IDB) and (B), (D), (F) for cyclic polyamide + [Cu(IDB)OOH]^+^-d(CGCTAACAGGC)_2 _(CPD^*γ*^-IDB)**.

For the [Cu(IDB)OOH]^+ ^ligand, the average RMSD values derived from the simulations over 12500 structures in the computed trajectories for HPD-IDB and CPD^*γ*^-IDB are 2.70 Å and 2.40 Å, respectively, as shown in Figures 3A and 3B. It has been tested that the mean-square fluctuations correlated with temperature factors for the systems are small, which predicts that the systems have attained simulation equilibriums. Figures 3C and 3D show the first sharp RDF peak, centered at 2.85 Å and 6.05Å, corresponding to direct and nonexistent interaction of OOH^- ^with the C4'H atom of sugar for HPD-IDB and CPD^*γ*^-IDB, respectively. In addition, Figures 3E and 3F present the probability distributions of O_d_-C4'H distance less than 3.5 Å with 34.7% of the simulation time versus those of the O_d_-C1'H distance less than 3.5 Å with 0% of the simulation time for HPD-IDB, and the probability distributions of the O_d_-sugar hydrogen atom distances of less than 3.5 Å all with 0% of the simulation time for CPD^*γ*^-IDB, respectively. The integration plots of probabilities in Figure 3G show that the C4'H abstraction is more favorable than the C1'H one for HPD-IDB. However, Figure 3F shows the properties of poor hydrogen abstractions for CPD^*γ*^-IDB.

**Figure 4:**
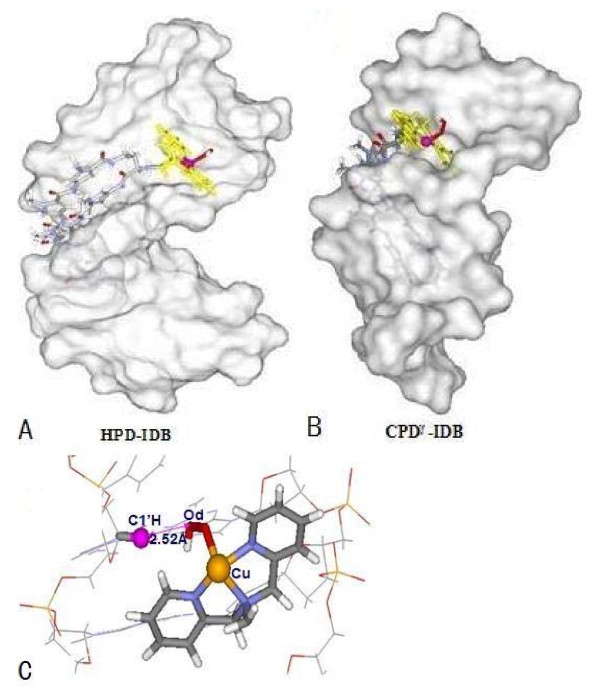
**Space-filling DNA models of polyamide + [Cu(IDB)OOH]^+ ^ligand binding to the minor groove of DNA; (A) for hairpin polyamide + [Cu(IDB)OOH]^+^-d(AATATCCACCTGCA)_2 _(HPD-IDB); (B) for cyclic polyamide + [Cu(IDB)OOH]^+ ^-d(CGCTAACAGGC)_2 _(CPD^*γ*^-IDB)**. **(C); average structure of hairpin polyamide + [Cu(BPA)OOH]^+ ^ligand orientating to C4'H atom of sugar.**

The average structure of each trajectory obtained from the corresponding simulation supports the general observations described above. The equatorial plane of [Cu(IDB)]^2+ ^nuclease in HPD-IDB is located perpendicularly at the wall of the minor groove of DNA with the angle of 80° between the plane of nuclease and the wall of minor groove, as shown in Figure 4A. However, for CPD^*γ*^-IDB as shown in Figure 4B, the [Cu(IDB)OOH]^+ ^ligand cannot be located at the minor groove of DNA, whose orientation is away from the minor groove of DNA, resulting from the rigidity of the N-tail of cyclic polyamide constraining the flexibility of [Cu(IDB) OOH]^+ ^ligand. This observation is similar to the properties of the cleavage agent of [Cu(BPA)OOH]^+ ^discussed above. There are also H-bonds formed between the N-H groups nearby Im/Py from the polyamides and the N or O atoms of nucleobases located on the floor of the minor groove of DNA (i.e., C...N distance of 2.52 Å), and between the H atom of the branch C atom in [Cu(IDB)]^2+ ^and the N/O atom of neighboring bases of DNA (i.e., C...O distance of 3.05 Å ). The average structures of HPD-IDB and CPD^*γ*^-IDB are shown in Figures 4A and 4B. The average structure from the simulation for HPD-IDB, as shown in Figure 4C, predicts the DNA cleavage possibility at the tail region of the polyamide, resulting from the Cu atom of [Cu(BPA)]^2+ ^approaching the nearest sugar C4'H of C10 base with the average O_d_-C4'H distance of 2.75 Å. However, it is obvious that the structural characteristics of CPD^*γ*^-IDB reveal theoretically the poor cleavage ability of [Cu(IDB)OOH]^+ ^ligand, which is similar to that of [Cu(BPA)OOH]^+ ^ligand discussed above.

Based on the discussion above, the two systems of HPD-BPA and HPD-IDB with different nuclease cleavage agents present similar cleavage properties to DNA. Namely, the [Cu(BPA)OOH]^+ ^and [Cu(IDB)OOH]^+ ^nucleases can be stably located at the minor groove of DNA, though the orientation of each cleavage agent in the minor groove of DNA is different. The [Cu(BPA)OOH]^+ ^nuclease is parallel to the wall of the minor groove of DNA, and the [Cu(IDB)OOH]^+ ^nuclease is located perpendicularly at the minor groove of DNA. On the other hand, for hairpin-polyamide recognition, the probability of hydrogen abstraction of the copper nuclease ligand [Cu(BPA) OOH]^+ ^from DNA is similar to that of the [Cu(IDB)OOH]^+ ^ligand, i.e., the C4'H abstraction from the sugar of DNA is favorable. However, for the two systems of CPD^*γ*^-BPA and CPD^*γ*^-IDB, both nuclease ligands cannot cause DNA strand scissions due to the inaccessibility of any hydrogen of the sugar moiety of DNA, which suggests that the DNA cleavage properties of [Cu(BPA)]^2+ ^and [Cu(IDB)]^2+ ^nucleases assisted by the cyclic polyamide are poor. In addition, though the recognition property of the cyclic polyamide with DNA is better than that of the hairpin polyamide,[48,53,56] the [Cu(BPA)]^2+ ^or [Cu(IDB)]^2+ ^nuclease assisted by the cyclic polyamide cannot achieve the specific DNA strand scission.

### Simulations of modified-cyclic-polyamide + [Cu(BPA)OOH]^+^/[Cu(IDB)OOH]^+^-DNA complexes (CPD^*γ*^Tail-BPA and CPD^*γ*^Tail -IDB)

**Figure 5:**
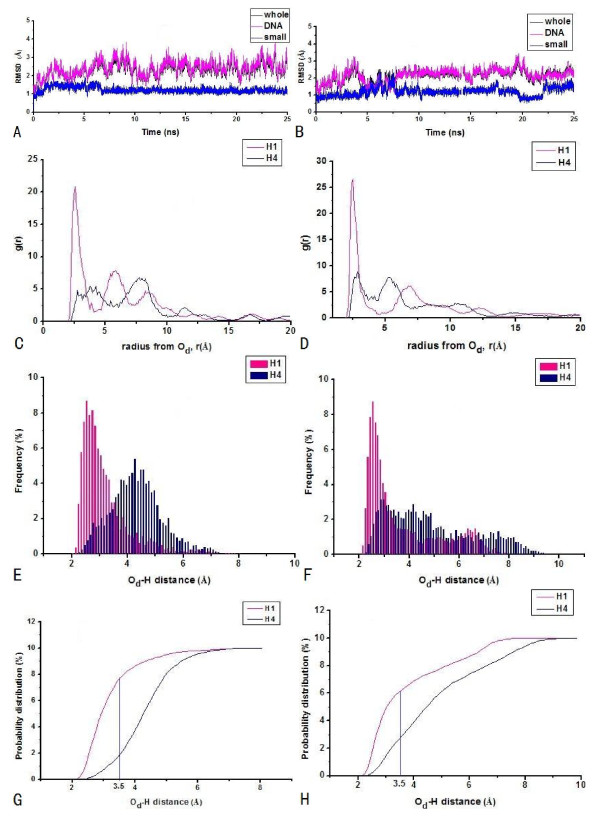
RMSDs with respect to the starting structures in the simulations of modified polyamide + copper nuclease -DNA (polyamide + copper nuclease -DNA (black), polyamide + copper nuclease (blue), and DNA alone (magenta)), RDF curves of the distal O_d _atom of OOH-C1'H atom (pink)/C4'H atom (navy) of sugars, probability distributions (the histogram bin width of 0.1 Å), and integration plots of probabilities of O_d_-C1'H (pink) and O_d_-C4'H (navy) distances: (A), (C), (E), (G) for modified cyclic polyamide + [Cu(BPA)OOH]+ -d(AATATCCACCTGCA)_2_ (CPDγTail-BPA) and (B), (D), (F), (H) for modified cyclic polyamide + [Cu(IDB)OOH]^+^-d(CGCTAACAGGC)_2 _(CPD^*γ*^Tail-IDB).

The discussions above indicate that the nuclease ligands for both systems of CPD^*γ*^-BPA and CPD^*γ*^-IDB cannot cause any hydrogen abstractions from the sugar moiety of DNA due to the rigidity of the N-tail of cyclic polyamide. It is obvious that the linker between the branch C atom of nuclease and the backbone of polyamide is very important for specific cleavage of metal nucleases to DNA. To improve the selective cleavage properties of metal nucleases assisted by cyclic polyamide, i.e., to increase the possibility of hydrogen abstraction from the sugar moiety, the flexible linker of -CH_2_CH_2_CH_2_NH_2 _was introduced to the cyclic polyamide in order to form the modified cyclic polyamide bound by each of the two nucleases, as shown in the additional files 1E and 1F. The corresponding complexes, modified-cyclic-polyamide + [Cu(BPA)OOH] ^+^/[Cu(IDB)OOH] ^+ ^- DNA, assigned as CPD^*γ*^Tail-BPA and CPD^*γ*^Tail-IDB, are built for the simulation calculations. The analyses of RMSDs of the two systems with respect to their initial structures, RDFs, probability distributions and integration plots of probabilities of O_d_-C1'H and O_d _-C4'H distances of the nearest bases (i.e., the cytosine in CPD^*γ*^Tail-BPA and the thymine in CPD^*γ*^Tail-IDB) are shown in Figures 5A, C, E and 5G for CPD^*γ*^Tail-BPA and Figures 5B, D, F and 5H for CPD^*γ*^Tail-IDB, respectively. Figures 5A and 5B show that the simulations initiated from the docked structures correspond closely to the average structures for both CPD^*γ*^Tail-BPA and CPD^*γ*^Tail-IDB systems. The small RMSD values predict that the interaction between the polyamide ligand and DNA molecule in each system is strong enough to hold the small [Cu(BPA)OOH]^+ ^and [Cu(IDB)OOH]^+ ^ligands in the minor groove of DNA. The average RMSDs of two systems derived from the simulations over 12500 structures in the computed trajectories for CPD^*γ*^Tail-BPA and CPD^*γ*^Tail-IDB are respective 2.27 and 2.14 Å (see Figures 5A and 5B), which supports that each system reaches a relatively stable situation during the course of the simulation. Figures 5C and 5D show the first sharp RDF peaks centered at 2.65 Å and 2.58 Å corresponding to direct interactions of O_d _atoms of OOH^- ^ligands with the C1'H atoms of sugars for CPD^*γ*^Tail-BPA and CPD^*γ*^Tail-IDB, respectively. In addition, Figure 5E presents the probability distributions of the O_d _-C1'H and O_d_-C4'H distances less than 3.5 Å with 72.3% and 15.8% of the simulation time for CPD^*γ*^Tail-BPA, respectively. Figure 5F presents the probability distributions of the O_d _-C1'H and O_d_-C4'H distances less than 3.5 Å with 62.5% and 25.2% of the simulation time for CPD^*γ*^Tail-IDB, respectively. The integration plots of probabilities in Figures 5G and 5H show that the numbers of C1'H atoms presented around O_d_-H distance of 3.5 Å are more than the numbers of C4'H atoms for these two systems. The results of probability distributions indicate that C1'H abstractions are favorable for both CPD^*γ*^Tail-BPA and CPD^*γ*^Tail-IDB systems. However, the possibility of C4'H or other sugar hydrogen atom abstractions is quite small.

**Figure 6:**
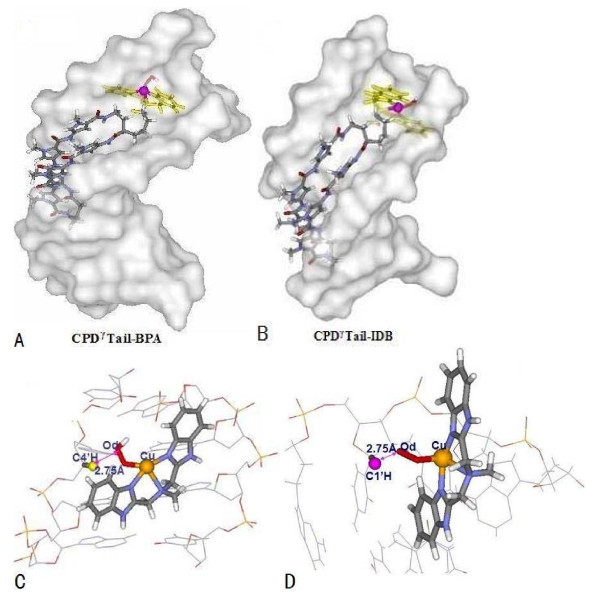
**Space-filling DNA model of [Cu(BPA)OOH]^+^/[Cu(IDB)OOH]^+ ^binding to the minor groove of DNA and average structures of [Cu(BPA)OOH]^+^/[Cu(IDB)OOH]^+ ^ligand orientating to C1'H atoms of sugars: (A), (C) for modified cyclic polyamide + [Cu(BPA)OOH]^+ ^-d(AATATCCACCTGCA)_2 _(CPD^*γ*^Tail-BPA) and (B), (D) for modified cyclic polyamide + [Cu(IDB)OOH]^+^-d(CGCTAACAGGC)_2 _(CPD^*γ*^Tail-IDB)**.

Visual analyses of the average structures obtained from trajectories of CPD^*γ*^Tail-BPA and CPD^*γ*^Tail-IDB reveal spatial details of the interactions of the entire ligands with DNA molecules, which supports the general observations described above. Specifically, (1) the modified cyclic polyamide + [Cu(BPA)OOH]^+ ^ligand or the modified cyclic polyamide + [Cu(IDB)OOH]^+ ^ligand remains stable in the minor groove of the corresponding DNA molecule; (2) the equatorial plane of [Cu(BPA)]^2+ ^nuclease is located perpendicularly at the wall of the minor groove of DNA with the angle of 72° between the plane of nuclease and the wall of minor groove of DNA for CPD^*γ*^Tail-BPA (see Figure 6A). The corresponding plane in CPD^*γ*^Tail-IDB is parallel to the wall of the minor groove of DNA with the angle of 28° between them (see Figure 6B); (3) for CPD^*γ*^Tail-BPA, the [Cu(BPA)OOH]^+ ^ligand is located at the small region of C8/G8 dyad based on the connection of the branch C atom of [Cu(BPA)]^2+ ^with the tail N atom of $\text{cyclo}[-\gamma -\text{ImPyPy}{-}^{CH_{2}CH_{2}{\text{CH}}_{\text{2}}NH_{2}}\gamma -\text{PyPyPy}-]$. The position of the O_d _atom of [Cu(BPA) OOH]^+ ^is close to the nearest sugar C1'H of G8 base with the average O_d_-C1'H distance of 2.52 Å, as shown in Figure 6C. At the same time, H-bonds between the C-H groups of [Cu(BPA)OOH)]^+ ^and O4/N2 atoms of C8/G8 bases in DNA with C...O or C...N distance of about 3.12 Å are observed in the data analysis. For the simulation of CPD^*γ*^Tail-IDB, the O_d _atom of the [Cu(IDB)OOH]^+ ^ligand approaches the nearest sugar C1'H atom of T4 base at the tail of $\text{cyclo}[-\gamma -\text{ImPyPy}{-}^{CH_{2}CH_{2}{\text{CH}}_{\text{2}}NH_{2}}\gamma -\text{PyPyPy}-]$ with the average O_d _-C1'H distance of 2.75 Å, as presented in Figure 6D. At the same time, H-bond events between the C-H groups of [Cu(IDB)OOH]^+ ^and O2/N2 atoms of T4/A4 bases in DNA occur with the C...O or C...N distance of about 3.25 Å.

In summary, the simulated results of CPD^*γ*^Tail-BPA and CPD^*γ*^Tail-IDB present the stable conformations of the [Cu(BPA)OOH]^+ ^and [Cu(IDB)OOH]^+ ^ligands respectively bound to the N-tail of the modified cyclic polyamide associated with DNA. With the -CH_2_CH_2_CH_2_NH_2 _linker introduced, the [Cu(BPA)OOH]^+ ^or[Cu(IDB)OOH]^+ ^ligand can stably lie in the minor groove of DNA. There are some contacts between the linker and the minor groove of DNA, which leads to the increase of binding affinity, resulting in enhancing the probability of DNA strand scission. These observations suggest that a flexibility linker between the recognition and cleavage agent can provide crucial contributions to specific DNA cleavage of metal nucleases. In addition, it can be seen from the comparison of the HPD-BPA(IDB) and CPD^*γ*^Tail-BPA(IDB) systems, the two nucleases assisted by both hairpin polyamide and modified cyclic polyamide with a linker -CH_2_CH_2_CH_2_NH_2 _all can achieve selective DNA cleavage via the hydrogen abstraction from the sugar moiety of DNA, although the abstracted hydrogen type is different, i.e., the nucleases bound to the hairpin polyamide can efficiently abstract the C4'H atom of sugar; however, the nucleases bound to the modified cyclic polyamide with a linker -CH_2_CH_2_CH_2_NH_2 _can efficiently abstract the C1'H atom of sugar. That is to say the chemical nucleases bound to an appropriate polyamide-type recognition agent can improve efficiently the DNA cleavage selectivity.

## Conclusion

A series of molecular dynamics simulations was performed to investigate the DNA cleavage specificities of the nucleases [Cu(BPA)]^2+ ^and [Cu(IDB)]^2+ ^assisted by the DNA recognition agents, hairpin and cyclic polyamides, via the analysis of hydrogen atom abstractions from the sugar rings in DNA. The simulated results indicate that the studied polyamide bound by the nuclease [Cu(BPA)]^2+ ^or [Cu(IDB)]^2+^, presents a stable conformation located in the minor groove of DNA, which is consistent with the conformation of the corresponding polyamide bound to DNA presented in X-ray structure. For the different nucleases assisted by hairpin polyamide in the two systems of hairpin-polyamide + [Cu(BPA)OOH] ^+ ^- DNA and hairpin-polyamide + [Cu(IDB)OOH] ^+ ^- DNA, the nucleases present the similar DNA cleavage properties. That is to say, the [Cu(BPA) ]^2+ ^or [Cu(IDB) ]^2+ ^nuclease with a substrate OOH^- ^bound to the hairpin polyamide can be stably located at the minor groove of DNA, and possibly abstracts the C4'H atom from the sugar moiety of DNA. However, for the [Cu(BPA)]^2+ ^or [Cu(BPA)]^2+ ^nuclease assisted with the cyclic polyamide in the systems of cyclic-polyamide + [Cu(BPA)OOH] ^+ ^- DNA and cyclic-polyamide + [Cu(IDB)OOH] ^+ ^- DNA, the probabilities of any hydrogen abstraction of the two nucleases from the sugar moiety of DNA are very small, which suggests that the nucleases cannot achieve DNA strand scission, due to the short and rigid linker between the branch C atom of nucleases and backbone of the cyclic polyamide. Therefore, the flexible linker of -CH_2_CH_2_CH_2_NH_2 _of *γ *region was introduced to the cyclic polyamide to improve DNA cleavage selectivity of the nucleases. It is of note that with the flexible linker introduction, the simulated results for the systems of modified-cyclic-polyamide + [Cu(BPA)OOH] ^+ ^- DNA and modified-cyclic-polyamide + [Cu(IDB)OOH] ^+ ^- DNA exhibit the high possibility of DNA cleavage by the [Cu(BPA)]^2+ ^or [Cu(IDB)]^2+ ^nuclease bound at the tail of the linker of the modified cyclic polyamide via C1'H atom abstraction from the sugar ring in DNA. Current observations suggest that the polyamide-type recognition agents with a flexible linker (*γ*-amino butyric acid) can efficiently improve the DNA cleavage properties of copper nucleases.

## Authors' contributions

HY carried out the computation and the analysis of data, and help in drafting the manuscript. YZ participated in the analysis of data and drafted the manuscript. YW conceived this work and critically revised the manuscript. GC have made substantial contributions to the interpretation and evaluation of the results, and helped in construction of the manuscript. We also wish to thank the advice of our reviewers. All authors read and approved the final manuscript.

## Supplementary Material

Additional file 1 **The component sketches of six polyamide + copper nuclease + OOH^- ^ligands**. The component sketches give the schemes of compositions and structures of hairpin polyamide + [Cu(BPA)OOH]^+ ^(A), hairpin polyamide + [Cu(IDB)OOH]^+ ^(B), cyclic polyamide + [Cu(BPA)OOH]^+ ^(C), cyclic polyamide + [Cu(IDB)OOH]^+ ^(D), modified cyclic polyamide + [Cu(BPA)OOH]^+ ^(E) and modified cyclic polyamide + [Cu(IDB)OOH] ^+ ^(F). Click here for file
