# Supplementary figures and images for: Investigation and improvement of DNA cleavage models of polyamide + Cu(II) nuclease + OOH- ligands bound to DNA
Source: BMC Struct Biol. 2010 Oct 17;10:35. doi: 10.1186/1472-6807-10-35 (PMC2984560; doi:10.1186/1472-6807-10-35)

**Additional file 1**


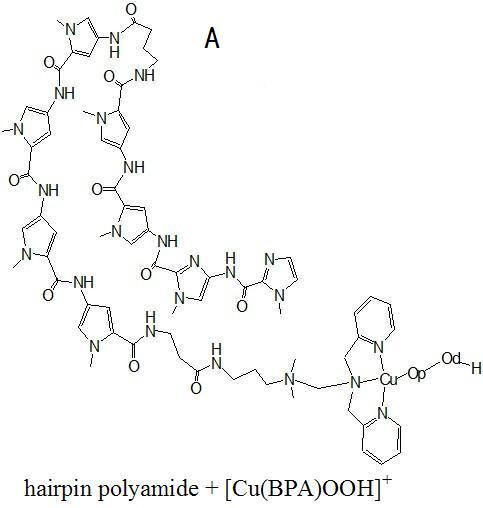

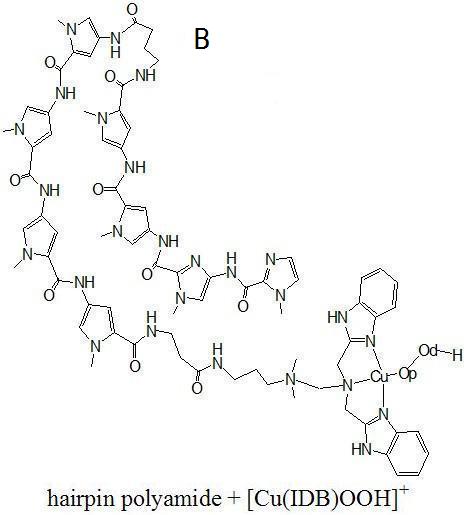


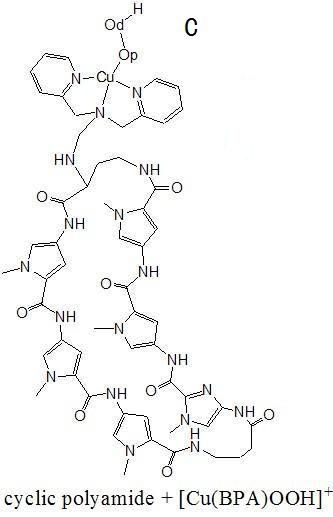

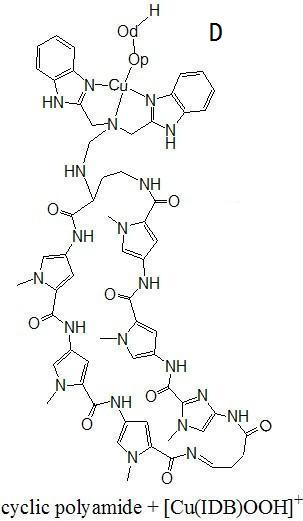


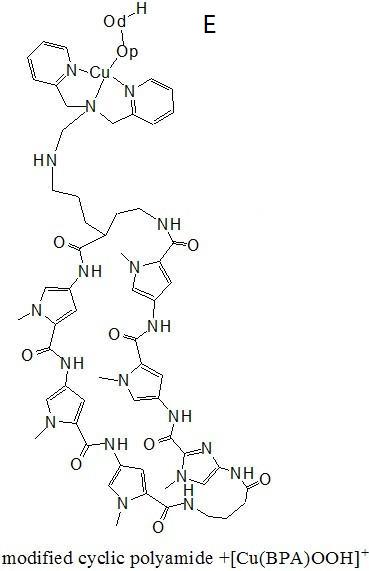

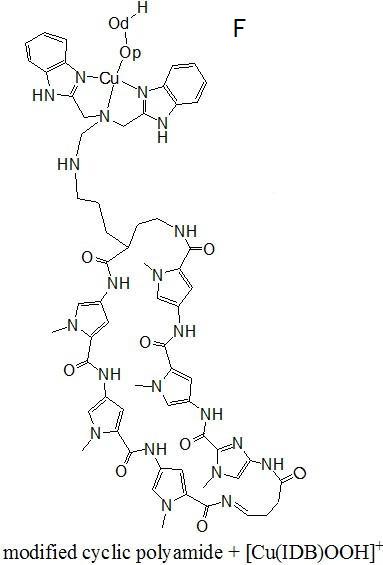

Supplement: Additional file 1 — The component sketches of six polyamide + copper nuclease + OOH- ligands. The component sketches give the schemes of compositions and structures of hairpin polyamide + [Cu(BPA)OOH]+ (A), hairpin polyamide + [Cu(IDB)OOH]+ (B), cyclic polyamide + [Cu(BPA)OOH]+ (C), cyclic polyamide + [Cu(IDB)OOH]+ (D), modified cyclic polyamide + [Cu(BPA)OOH]+ (E) and modified cyclic polyamide + [Cu(IDB)OOH] + (F). [file 1472-6807-10-35-S1.DOC]
